# Crystal structure of the mitochondrial protein mitoNEET bound to a benze-sulfonide ligand

**DOI:** 10.1038/s42004-019-0172-x

**Published:** 2019-07-03

**Authors:** Werner J. Geldenhuys, Timothy E. Long, Pushkar Saralkar, Toshio Iwasaki, Raisa A.A. Nuñez, Rajesh R. Nair, Mary E. Konkle, Michael A. Menze, Mark V. Pinti, John M. Hollander, Lori A. Hazlehurst, Aaron R. Robart

**Affiliations:** 1 Department of Pharmaceutical Sciences, School of Pharmacy West Virginia University, Morgantown, WV 26506, USA.; 2 Department of Pharmaceutical Sciences and Research, School of Pharmacy, Marshall University, Huntington, WV 113-8602, USA.; 3 Department of Biochemistry and Molecular Biology, Nippon Medical School, Sendagi, Tokyo 113-8602, Japan.; 4 Department of Biochemistry, School of Medicine West Virginia University, Morgantown, WV 26506, USA.; 5 Department of Microbiology, School of Medicine West Virginia University, Morgantown, WV 26506, USA.; 6 Department of Chemistry, Ball State University, Muncie, IN 47306, USA.; 7 Department of Biology, University of Louisville, Louisville, KY 40292, USA.; 8 Department of Physiology, School of Medicine West Virginia University, Morgantown, WV 26506, USA.; 9 Modulation Therapeutics, Morgantown, WV 26506, USA.; 10 These authors contributed equally: Werner J. Geldenhuys, Timothy E. Long, Pushkar Saralkar.

## Abstract

MitoNEET (gene *cisd1*) is a mitochondrial outer membrane [2Fe-2S] protein and is a potential drug target in several metabolic diseases. Previous studies have demonstrated that mitoNEET functions as a redox-active and pH-sensing protein that regulates mitochondrial metabolism, although the structural basis of the potential drug binding site(s) remains elusive. Here we report the crystal structure of the soluble domain of human mitoNEET with a sulfonamide ligand, furosemide. Exploration of the high-resolution crystal structure is used to design mitoNEET binding molecules in a pilot study of molecular probes for use in future development of mitochondrial targeted therapies for a wide variety of metabolic diseases, including obesity, diabetes and neurodegenerative diseases such as Alzheimer’s and Parkinson’s disease.

MitoNEET (gene *cisd1*) was identified in 2004 as the first example of a mitochondrial outer-membrane iron–sulfur protein, with the cluster binding domain facing the cytosolic side^1^. This protein was initially discovered as the unintended target for the anti-diabetic peroxisome proliferator-activated receptor gamma (PPAR-γ) agonist pioglitazone, as many of the beneficial effects of pioglitazone, a thiazolidinedione (TZD), could not be explained by PPAR-γ activity alone^1^. MitoNEET assembles as a homodimer, with each subunit containing a rather unusual [2Fe–2S]-type iron–sulfur cluster coordinated by His87 and three cysteine residues^2^. The amino acids coordinating the [2Fe–2S] cluster form a signature CDGSH domain common to all members of the CISD-gene family. Bioinformatics analysis has shown that the CISD-gene family is highly conserved across a variety of species, including mammals, *Caenorhabditis elegans*, plants as well as thermophilic archaea and bacteria, with a high degree of structural homology (particularly around the cluster binding domain) among the different phyla^3^. To date three CISD proteins have been crystalized, including human mitoNEET (gene *cisd1*), miner1/NAF-1 (gene *cisd2*), and miner2/miNT (gene cisd3)^4,5^.

MitoNEET plays an important role in mitochondrial function and metabolism^6–10^. Overexpression of mitoNEET in the adipose tissue of *ob*/*ob* mice led to a significant reduction in inflammation and oxidative stress as compared to the control mice^7^. In addition, overexpression of mitoNEET in cardiomyocytes was protective against oxidative stress as induced by hydrogen peroxide^11,12^. In contrast, when mitoNEET was knocked out of mice the resulting phenotype was characterized by a loss of dopamine neurotransmitter levels from the striatum and Parkinson’s disease type motor deficits^13^. Although the detailed mechanistic aspects underlying these physiological functions remain elusive, current understanding of mitoNEET correlates to the crucial role of the redox-active [2Fe–2S] clusters, possibly serving as an outer-membrane redox-sensor and pH sensor for mitochondrial function and/or a potential source of the iron–sulfur cluster transfer to cytosol in response to the redox states in the cells^8,9^.

Despite the fact that mitoNEET was described relatively recently, numerous crystal structures have been reported for both the wild-type and mutants of the soluble portions (PDB codes 2QH7, 2QDO, 2R13, 3EW0, 3REE, 3LPQ, 4EZF, 4F1E, 4F28, and 4F2C). However, none of these crystal structures includes a bound ligand that has drug-like activity. Here, we describe the crystal structure of a mitoNEET–ligand complex with furosemide. This structure can be used to gain structural insights into mitoNEET binding to ligands, as well as be used in structure-based drug discovery (SBDD) studies.

## Results

### Co-crystallization study.

Based on the published literature, mitoNEET represents a potential drug target for the development of compounds to treat a variety of metabolic diseases^6,7,14^. Unfortunately, no crystal structure has been solved with a ligand bound to the protein for use in SBDD studies. Here, we present the first crystal structure at 1.95 Å resolution of human mitoNEET with a bound benzoic-sulfonamide-furan ligand, furosemide. Our previous approaches to screening condition for soaking with the prototypical mitoNEET ligands TZDs such as pioglitazone and NL-1 did not yield any crystals, mainly due to solubility problems of the TZD-containing compounds. This led us to explore an alternative approach, which was to find a soluble mitoNEET ligand which is suitable for co-crystallization with the human mitoNEET protein. We theorized from earlier crystal structure of mitoNEET that the LYS55 and HIS87 would be of importance, and the free carboxylic group of furosemide would likely interact via hydrogen bonding^15^. We have previously reported that furosemide binds to mitoNEET with moderate affinity (IC_50_ ~40 μM)^16^, using a recombinant mitoNEET with a mitoNEET-His protein, as opposed to the His-SUMO protein we used here for crystallography. We focused on furosemide in this study due to its appropriateness for the X-ray diffraction studies, due to its favorable solubility profile, as opposed to the glitazones where solubility in the crystallization mother liquor is limiting. In addition, we did not observe any aggregate formation with furosemide in solution using dynamic light scattering (DLS), which could interfere with the crystallography studies (Supplementary Fig. 1).

**Fig. 1:**
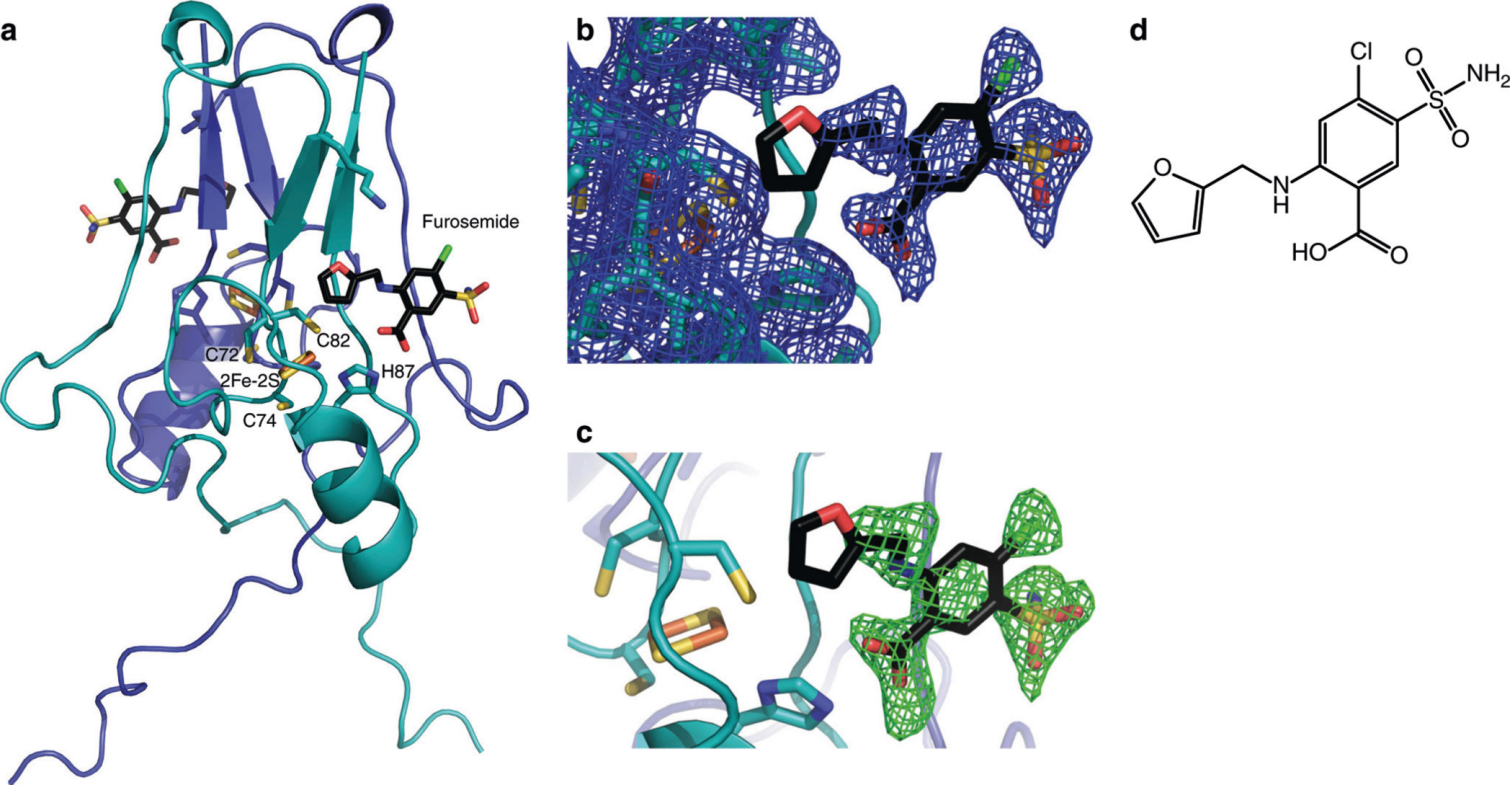
Crystal structure of human mitoNEET in complex with the bound ligand furosemide. **a** MitoNEET dimer shown with the [2Fe–2S] cluster and coordinating amino acids. Furosemide is shown in atom colors. **b** *2Fo*–*Fc* map (contoured at 1*σ*) showing bound ligand electron density. **c** *Fo*–*Fc* OMIT map (contoured at 2.5*σ*) further validates the position of the bound furosemide ligand. **d** Structure of furosemide

The crystal structure of furosemide bound to mitoNEET is shown in Fig. 1, with the crystal data (6DE9.pdb) given in Table 1. In the refined structure, furosemide is bound on the face close to the [2Fe–2S] cluster in each monomer, which are critical for the biological function of mitoNEET (Fig. 1a). The bound ligand was supported by strong density after MR-SAD phasing and refinement, and was further confirmed by simulated annealing OMIT map analysis (Fig. 1b, c). The furosemide carboxylic groups are in reach of the [2Fe–2S] coordinating His87 side chain for possible hydrogen bonding with His87Nε. In addition, the furan ring of furosemide can be modeled such that the oxygen is pointed toward Sγ of the Cys83 ligand, although the limited resolution of the refined electron density map cannot discriminate the precise orientation of the furosemide furan ring in the present study. The furosemide-binding site observed in the crystal structure corresponds to previously hypothesized binding pocket, from molecular docking studies performed with the anticancer drug MAD-28 corroborating our finding^17^. In addition, the pocket identified by furosemide is suggested to correspond to a possible binding site for pioglitazone, for which nuclear magnetic resonance studies indicated causes perturbations in the overall structure and to be close to a Trp or Phe residues, although the exact residues still need to be determined^2^. In accordance, the Phe82 side chain is within a 5 Å distance from the furosemide furan ring in the refined structure, which could suggest that this is the same binding site that pioglitazone occupies. We previously identified other possible binding pockets from docking studies, which was located near the α-helix of mitoNEET, and predicted this to be the primary site for resveratrol-3-sulfate and pioglitazone binding^15,18^. Considering also the previous report that pioglitazone inhibits cluster release from mitoNEET under oxidative stress^19^, it seems likely that the compounds binding in the furosemide pocket would stabilize the ligation environment of the [2Fe–2S] cluster. The observation that mutation of HIS87 to CYS has a cluster stabilizing effect and caused a significant shift in the reduction potential is also consistent with this model^20^. Regardless of whether the main function of mitoNEET in the cell is as a redox sensor, iron chaperone, or as an electron-transport protein, binding of a ligand so near to the cluster is likely to have a significant impact on cellular energetics.

### Ligand development.

**Fig. 2:**
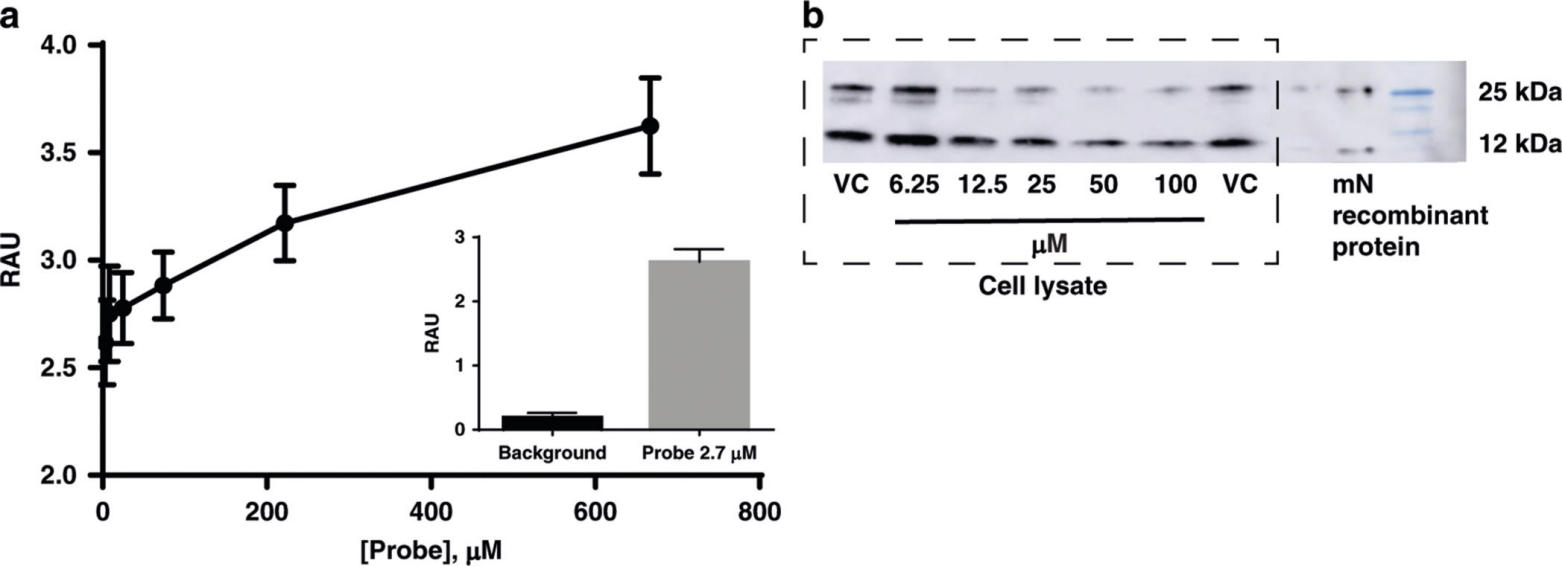
Interaction with furosemide and mitoNEET. **a** To evaluate an indirect binding effect of the interaction of furosemide with mitoNEET, a ELISA biotin–streptavidin capture assay shows that the furosemide–biotin probe is able to capture mitoNEET and be detected with a primary antibody; **b** the addition of furosemide (0–100 μM) is able to reduce the amount of mitoNEET captured from a N2A cell lysate by the furosemide-probe attached to streptavidin breads. Treatment: mitoNEET (mN); vehicle only (VC). RAU relative absorbance units

As proof of principle, we developed a small pilot set of compounds to validate that the interaction between furosemide and mitoNEET, both in the binding assay and the crystal structure. This was in part to ensure that the interaction we observe for furosemide was not due to nonspecific interaction with the [^3^H]rosiglitazone. We developed an ELISA capture assay using a streptavidin capture plate and a biotin–furosemide probe. As can be seen in Fig. 2a, mitoNEET recombinant protein is dose-dependently captured by the probe and is detectable by a primary antibody against mitoNEET, suggesting that the interaction we have observed from our binding assays were due to a binding interaction. Furthermore, we used the probe to isolate recombinant mitoNEET from a sample of cell lysate (Fig. 2b). In the presence of furosemide, we were able to show dose-dependently the reduction of mitoNEET captured in the pull-down assay. The most prominent reduction in binding was seen with the ~25 kDa band, in contrast to the ~12 kDa. Lastly, we used an orthogonal assay to assess interaction between mitoNEET and furosemide. Surface plasmon resonance (SPR) indicated that the association rate constant *K*_on_ was 5.6 × 10^2^ M^−1^ s^−1^, a dissociation rate constant *K*_off_ of 3.0 × 10^−2^ M^−1^ s^−1^ and the equilibrium dissociation constant *K*_d_ is 53.5 μM (Supplementary Fig. 2).

**Fig. 3:**
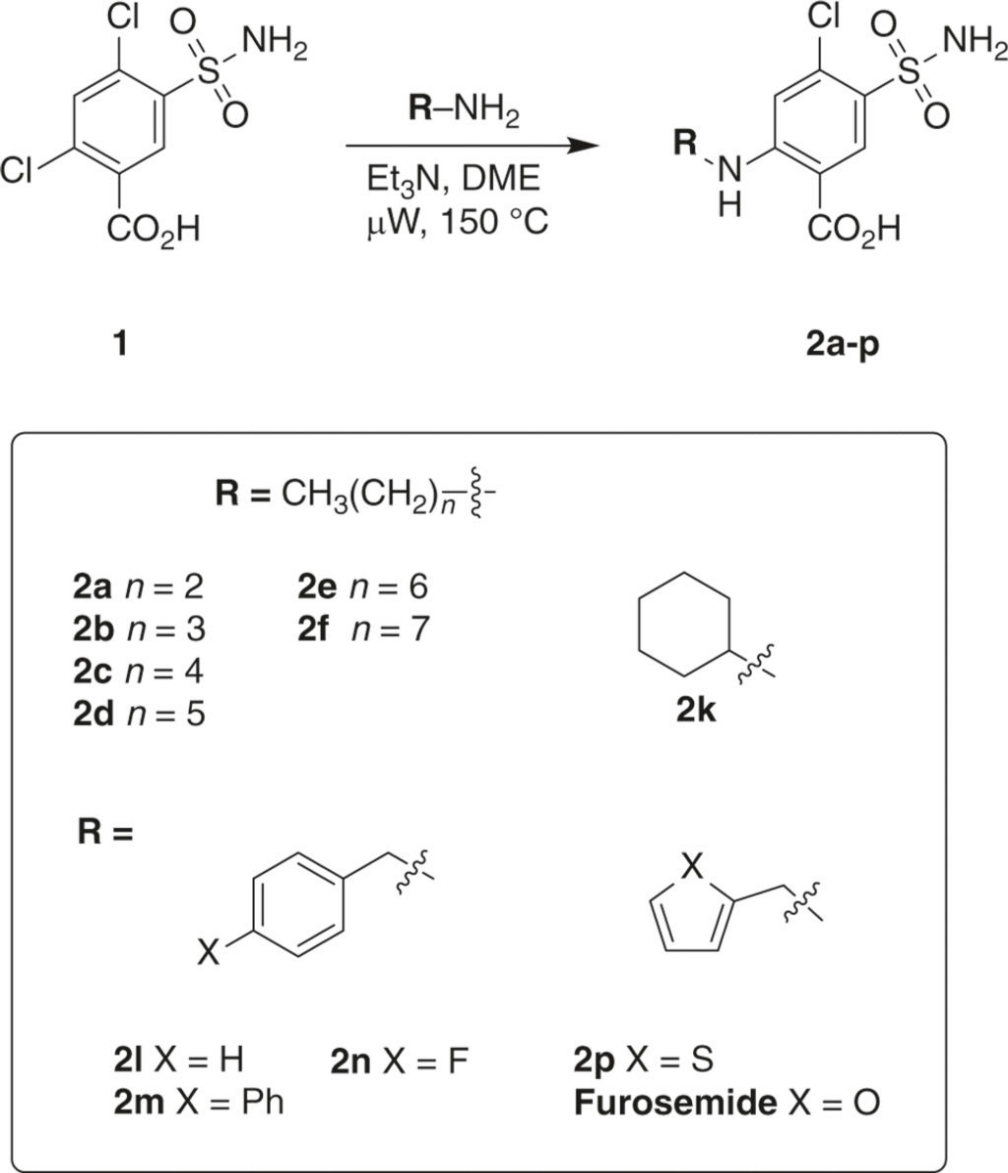
Microwave-assisted synthesis of furosemide analogs. Eleven compounds **2a–p** were prepared from the commercially available sulfonamide **1** by microwave synthesis and tested for mitoNEET binding and respiration studies

**Fig. 4:**
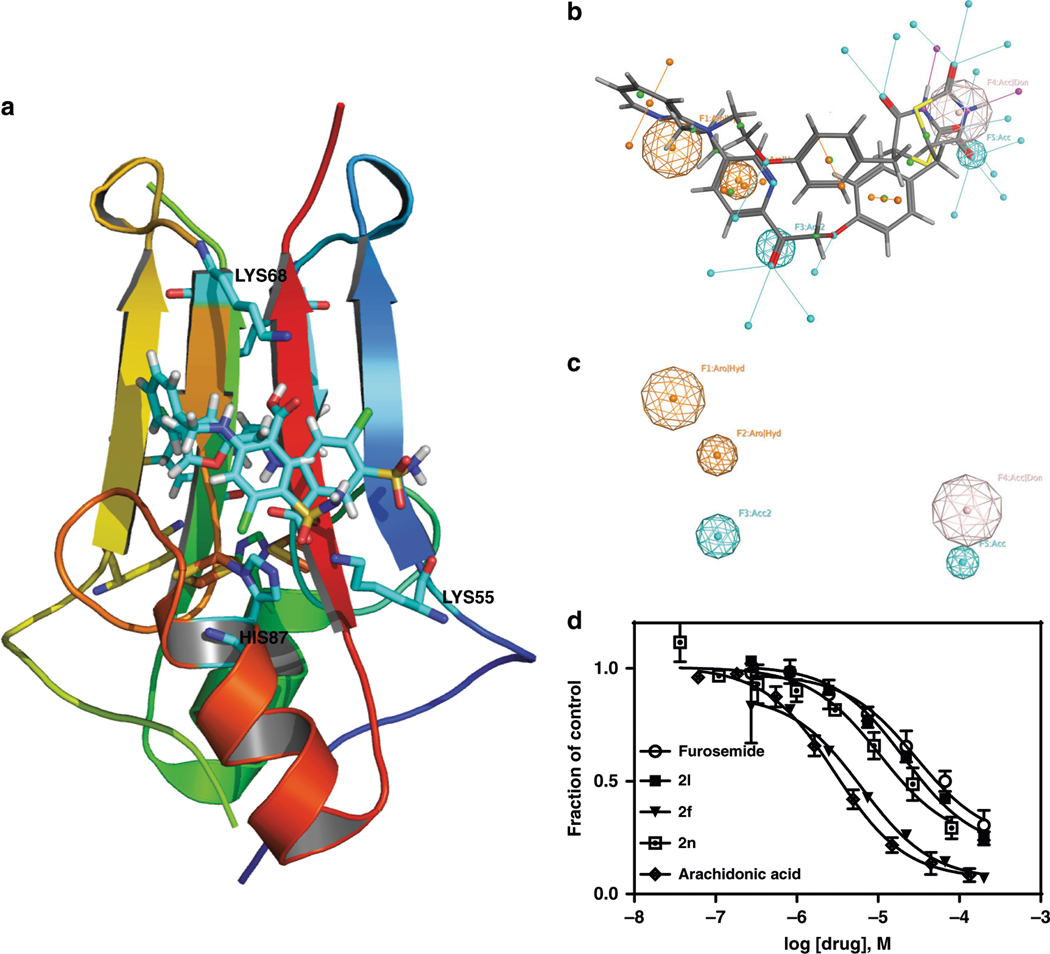
X-ray crystal of mitoNEET with furosemide used to develop novel derivatives. **a** Docked poses of compounds, **2f**, **2l**, and **2n** to illustrate the ability to design compound to interact with HIS87, LYS55, and LYS68; **b**, **c** Pharmacophore query for the binding pocket from docking studies. The furosemide bound mitoNEET structure was used for the docking study. **d** Binding curves of compounds in the presence of [3H]rosiglitazone

The discovery that we could use furosemide as a probe for crystallography of mitoNEET, led us to synthesize a series of derivatives to validate the use of our crystal in molecular probe development, and for exploring structure–activity relationships of these sulfonamide compounds. As proof of principle, we synthesized 11 furosemide analogs (Fig. 3 which were prepared from the commercially available sulfonamide **1** by microwave synthesis (see Supplementary methods)). Before we synthesized the compounds, we docked them into the furosemide-binding site, and found them to occupy the binding pocket supporting synthesis initiation. The displacement IC_50_s of [^3^H]rosiglitazone with the human recombinant mitoNEET for these compounds are shown in Table 2 and Fig. 4 showing representative binding curves. We found that the extension of the alkyl chain increased the affinity, with an optimal length of between five and eight carbons within the current set of compounds tested. The importance of an aliphatic or aromatic side chain was verified from the use of compound 1, which does not have either of these substitutions. Similarly, based on this observation, we found that arachidonic acid showed similar binding characteristics, sharing both the ring type structure of the TZD warhead and the lipophilic tail^21^. We found that inclusion of ring structures, specifically aromatic in nature, would improve the affinity of compounds. For example, the exclusion of a ring in **2a** led to a dramatic decrease in ability to displace [3H[rosiglitazone from observed IC_50_ of 22 μM of furosemide to 81 μM for **2a**.

A general pharmacophore model is shown in Fig. 4. Using the crystal structure of furosemide with mitoNEET, we docked a series of compounds, (furosemide, TT01001, NL-1, and rosiglitazone) into the furosemide-binding pocket/site, and developed a preliminary pharmacophore model for the pocket. In this case, pharmacophore model for mitoNEET ligands best described by the appearance of two hydrophobic/aromatic centers, two hydrogen bond acceptors, and one hydrogen bond acceptor/ donor, underpinning future studies into interactions with LYS55 and HIS87 similar to interactions suggested with other compounds such as the anticancer compound MAD-28^17^.

**Fig. 5:**
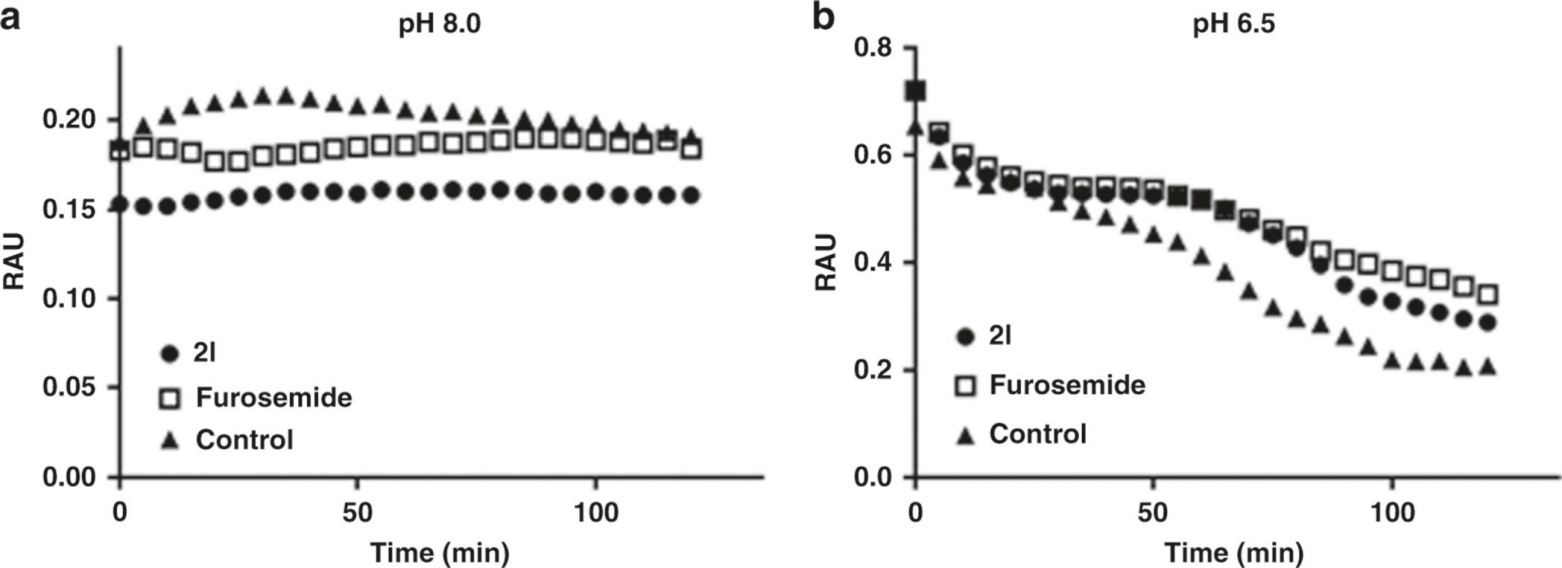
Iron–sulfur cluster release from mitoNEET. Furosemide is able to slow down cluster release comparing **a** pH 8 to **b** pH 6.5 when measured at 458 nm. Compounds were tested at 20 μM final concentration each. Vehicle control is mitoNEET (mN) with DMSO

Previous studies have shown that mitoNEET loses the iron–sulfur [2Fe-2S] clusters in low pH^22^. To validate that the interaction between furosemide and mitoNEET in the crystal is not due to nonspecific interactions, we tested furosemide’s ability to slow down the release of the clusters form mitoNEET. As seen in Fig. 5, furosemide is able to reduce cluster loss at pH 6.5 suggesting the interaction with mitoNEET to be specific to the cluster region.

**Fig. 6:**
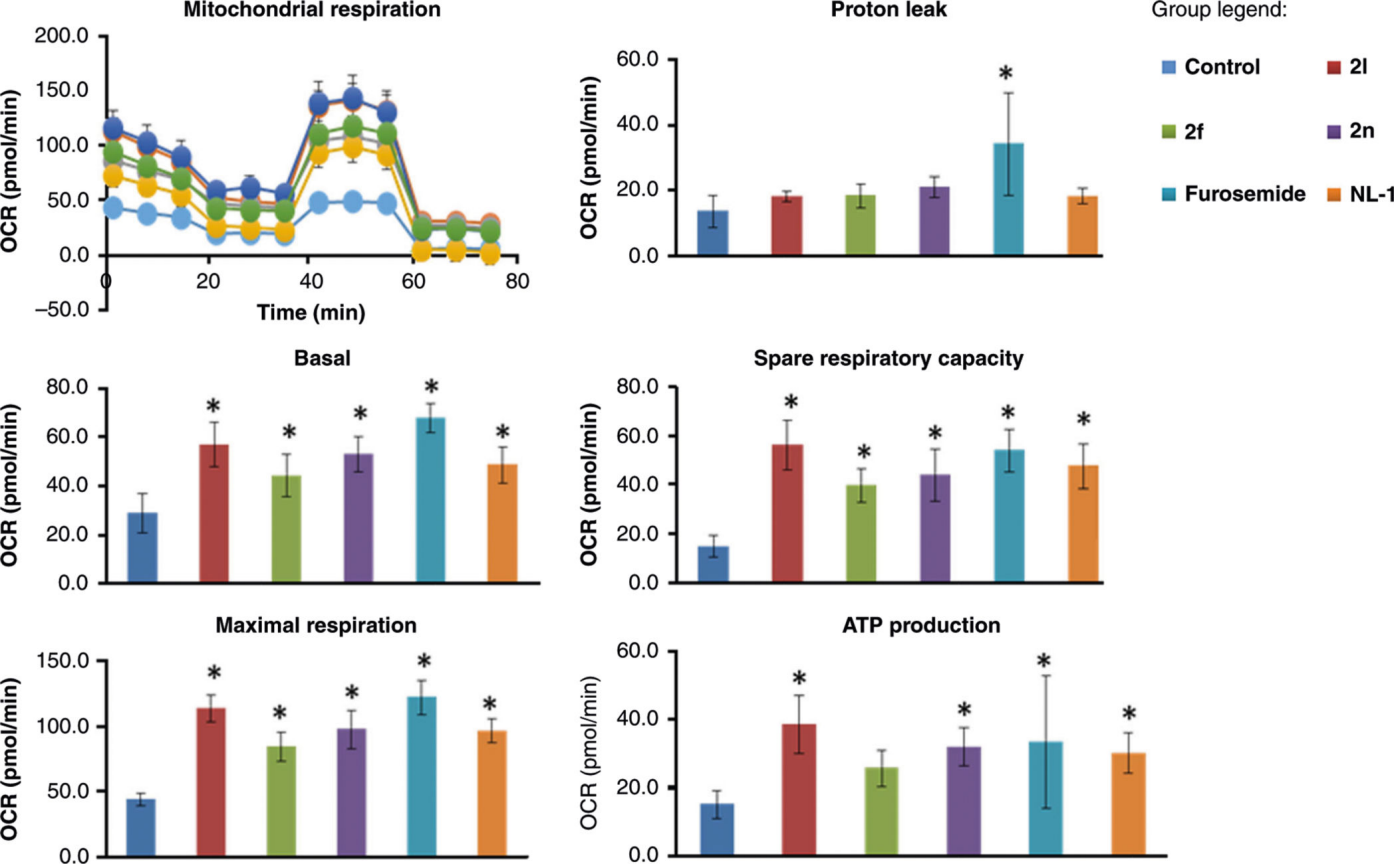
Mitochondrial effect of the compounds on murine neuronal N2A cells. The Seahorse Bio-analyzer was used to evaluate the effect of compounds on mitochondrial function and bioenergetics in cells. Compounds were tested at 20 μM, with vehicle control treatment with DMSO only. Each bar represents mean ± S.D. where *N* = 8. *Statistical significance *P* < 0.05

**Fig. 7:**
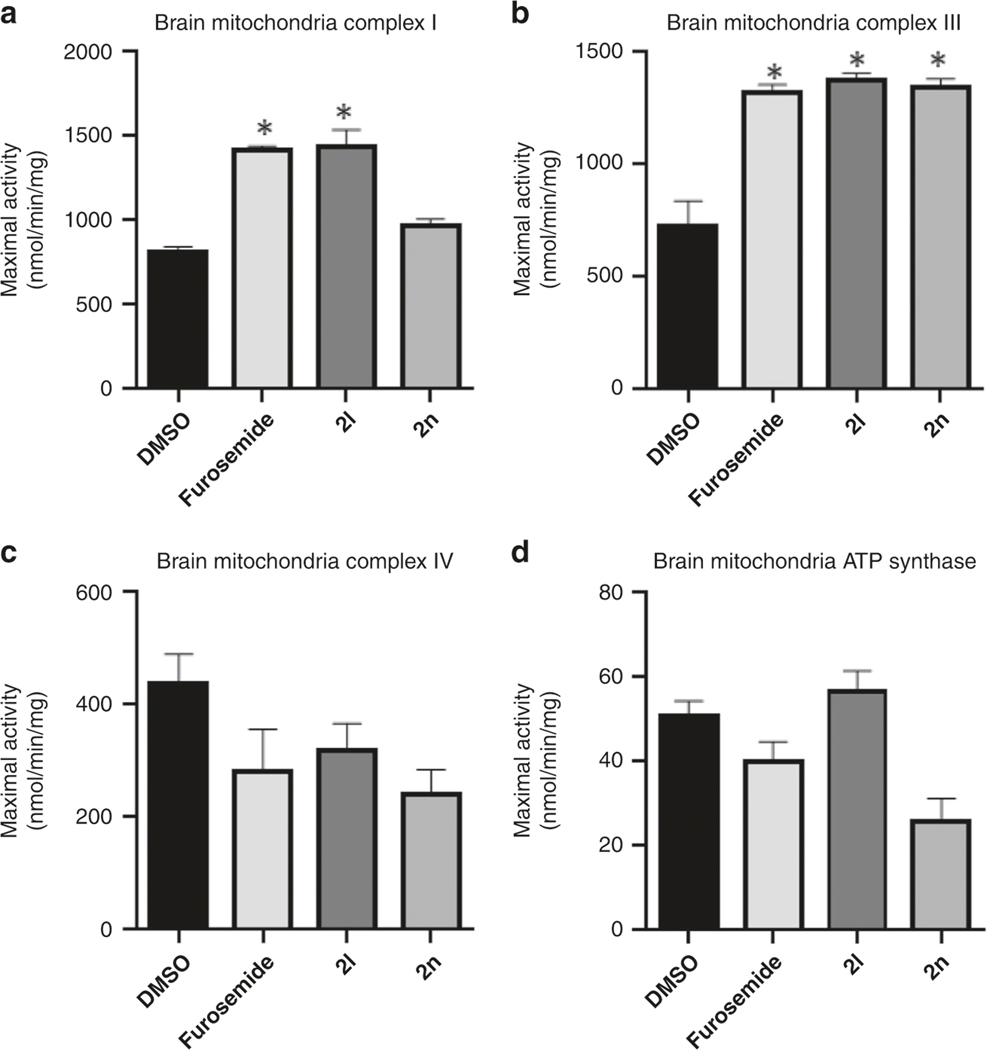
Electron-transport complex activity of isolated murine brain mitochondria. Complex activity of isolated brain mitochondria were affected by compounds. **a** Complex I; **b** Complex III; **c** Complex IV, and **d** ATP Synthase. Compounds were tested at 20 μM, with vehicle control treatment with DMSO only Each bar represents mean ± S.D. where *N* =6. Statistical significance *P < 0.05

Furosemide was chosen for this crystal structure study due to speculated interaction to key residues^9^, as well as previous literature which indicated a possible mitochondrial interaction^23^. Since we found that furosemide binds to mitoNEET, albeit in the micromolar range, and that mitoNEET is thought to regulate mitochondrial bioenergetics^2,17,24^, we evaluated the new derivatives for possible mitochondrial activity. To evaluate the new compounds for pharmacological activity, we tested their ability to affect mitochondrial bioenergetics (Fig. 6). N2A cells were used to evaluate the activity, since most of our previous work with mitoNEET centered on neurodegenerative diseases, including Parkinson’s disease^13^. As control compounds, we used furosemide and the mitoNEET ligand NL-1. We found that the compounds 2l (mitoNEET IC_50_ = 20.7 μM) and 2f (mitoNEET IC_50_ = 6.2 μM) were able to increase both basal respiration (oxygen consumption rate), and maximal respiration in each case, similar to NL-1 and furosemide. The spare respiratory capacity was also increased, with a commiserate increase in ATP-linked oxygen consumption. As signs of health, the proton leak and spare respiratory capacity were not changed with the addition of the compounds, although furosemide trended to increase proton leak. In support of these findings in cell culture, we isolated fresh brain mitochondria from mice. Evaluation of the oxidative phosphorylation complexes showed that furosemide interacts with mitochondria (Fig. 7), specifically we noticed an increase in complex I and III activity. Similar effects were seen when we tested two other compound, **2f** and **2l**. Complex IV and ATP synthase were mildly inhibited by the compounds at the concentration tested. DLS follow-up studies suggested that the compounds did not form aggregates in the buffers, which could lead to false positives (Supplementary Fig. 1)^25^. The current interpretation of the mitochondrial data in the presence of furosemide does not exclude that possibility that the effects could be unrelated to mitoNEET biochemistry. Future studies will be needed to evaluate the full scope of biochemical activity of furosemide at the mitochondrial level, as well as its phenotypical role in disease management.

## Discussion

MitoNEET is a mitochondrial protein with redox and pH sensor activity, which has been of interest toward designing compounds to modulate bioenergetics of mitochondria^2,17,22,24^. Of late no structure has been published which contains mitoNEET protein with a small organic compound bound as a complex, which has hampered progress in designing mitoNEET ligands. In this study, our goal was to identify a compound that could be used to achieve a co-crystal structure with mitoNEET, as the glitazones tended to precipitate out of solution failing to co-crystalize. We present a mitoNEET crystal structure in complex with a benzoic-sulfonamide-furan drug bound for use in future drug development studies. Our crystal structure was used to design compounds that could give insight into structure–activity relationships of the scaffold. The primary binding site identified correlates with the redox-active [2Fe–2S] cluster ligation site, and binding to the HIS87 and LYS55 may inhibit its redox function and/or possible cluster release/breakdown at the cytoplasmic side^2,17,24^. With the current results, discovery programs can be focused on developing probes to study the pharmacology of mitoNEET and possibly develop novel compounds as first-in-class to treat mitochondrial dysfunction such as Parkinson’s disease^26^.

## Methods

### Chemical synthesis.

See Supplementary methods.

### Protein expression and purification.

Plasmid pET11a-His-SUMO-mitoNEET was constructed from a synthetic gene block (IDT) cloned between Nde I and Bam HI. MitoNEET recombinant protein for crystallography was expressed as a fusion with His-SUMO. The sequence of the expressed protein was as follows: MSGHHHHHHHHGGGSGSSGGGSDSEVNQEAKPEVKPEVKPETHINLKVSDGSSEIFFKIKKTTPLRRLMEAFAKRQGKEMDSLRFLYDGIRIQADQTPEDLDMEDNDIIEAHRE QIGGTKRFYVKDHRNKAMINLHIQKDNPKIVHAFDMEDLGDKAVYCRCWR SKKFPFCDGAHTKHNEETGDNGPLIIKKKET.

Protein expression was performed in Rosetta 2 (DE3) cells. Cells were grown in TB both by autoinduction at 25 °C overnight, harvested at 5000 × g for 15 min, and resuspended in lysis buffer (20 mM Tris-HCl (pH 8.5), 0.3 M NaCl, 10 mM imidazole). Cells were lysed by sonication, and the lysates were cleared at 15,000×g for 15 min. Cleared lysate was bound to Ni-NTA resin (Gold Biotechnology), washed with lysis buffer containing 20 mM imidazole, and eluted with lysis buffer containing 300 mM imidazole. Purified mitoNEET was desalted over a PD-10 column with 10 mM Tris-HCl (pH 8.0), 100 mM NaCl. The purification tag was removed by digestion with His-tagged SUMO protease at 4 °C overnight. The cleaved tag and His-SUMO protease was removed by a second binding to Ni-NTA resin, followed by size exclusion chromatography. The His tag was followed by a flexible Ser/Gly linker fused to a SUMO motif. After expression SUMO protease (specific cleavage after QIGG) was used to remove the fusion purification tag. After these steps, the final amino acid primary sequence is as follows:

TKRFYVKDHRNKAMINLHIQKDNPKIVHAFDMEDLGDKAVYCRCWRSK KFPFCDGAHTKHNEETGDNVGPLIIKKKET.

### Crystallization and refinement of mitoNEET-furosemide.

Purified mitoNEET protein (20 mg/ml) mixed with an equal molar concentration of furosemide was screened by sitting drop vapor diffusion against Index screen (Hampton Research). Red crystals formed in under a week in several conditions. Final data collection was performed using crystals grown by sitting drop vapor diffusion by mixing a 1:1 ratio of protein with 60% v/v Tacsimate pH 7.0. The mother liquor was used for cryoprotection. Data collection was performed on the Northeastern Collaborative Access Team beamline 24-ID-C at the Advanced Photon Source at the iron peak. Data were indexed, integrated, and scaled using iMOSFLMand Aimless. To avoid model bias molecular replacement was combined with anomalous signals (MR-SAD) using Phaser and PDB 3REE as the search model^15^. Refinement was performed using phenix^27^. Model building/rebuilding was performed using Coot^28^. OMIT maps were constructed by removing the ligand from the final refined structure file, performing three macro-cycles of refinement with simulated annealing, and calculating a |*F*_o_|—|*F*_c_| map with phenix.maps.

### MitoNEET binding assay.

Recombinant mitoNEET was attached to nickel scintillation proximity assay (SPA) beads, and incubated with 20 nM [^3^H] rosiglitazone (PerkinElmer) for 60 min at room temperature. The binding buffer was 50 mM Tris pH 8. White clear bottom 96-well plates were used for the assay, and the counts per minute analyzed in a MicroBeta Counter (Perkin Elmer). Data were analyzed as fraction of the total binding using a one-site model with GrapPad Prism 6 statistical software. All binding curves had *R*^2^ > 0.9.

### Surface plasmon resonance.

SPR studies were done by the contract research organization CreativeBiolabs (https://www.creative-biolabs.com/). Experiments were performed using Reichert4SPR refractometer system (Reichert Technologies, Depew, NY, USA) equipped with a dextran SPR sensor chip (Reichert carboxymethyl dextran P/N 13206066). Acetate buffer was used for immobilization of the protein MN (ligand). First, the surface was stabilized with phosphate buffer saline (PBST; PBS with 0.05% Tween 20, pH7.4) at a flow rate of 25 μL/min (25 °C) until a constant refractive index was obtained. Carboxymethyl dextran on a chip was activated by injecting a solution of 10 mg/mL NHS and 40 mg/mL EDC over the sensor chip surface for 7 min at a flow rate of 10 μL/min. Then the ligand, 60 μg/mL in 10 mM sodium acetate (pH 4.5), was immobilized by injection over the surface for 7 min. The unreacted sites on the sensor chip surface were blocked by injection of 1 M ethanolamine (pH 8.5) for 8 min. A furosemide solution with 7 different concentrations (18.7, 25, 37.5, 50, 75,100, and 150 μM) prepared in PBST (PBS with 0.05% Tween 20, pH7.4) with 1% DMSO was injected into the cell over both channels at a flow of 25 μL/min, association for 1.5 min, dissociation for 2.5 min. Data analysis were performed with TraceDrawer software. Final *K*_d_ values were determined by fitting to a one-to-one binding model.

### Docking studies.

The docking studies were performed using Glide (Schrodinger.com). Our mitoNEET protein structure (6DE9) with furosemide was used in the docking studies. The structure was prepared for docking using the PrepWizard that added the necessary hydrogens. Glide SP docking was used in the default setting for the docking studies of each compound. The top resultant poses were visually inspected and the ligand interaction diagram function used to determine hydrogen bonding. Pharmacophore features were identified from the docked poses and the Pharmacophore editor in MOE 2018 (www.chemcomp.com). Final poses were visualized in PyMol version 0.99.

### Seahorse bioenergetics.

Mitochondrial bioenergetics were evaluated by the use of the Seahorse 96-well plate reader. Cells were grown in specialty plates from the company, at 20,000 cells per well of N2A mouse neuronal cells. The cells were treated with compounds at 20 μM for three hours, and then a mitochondrial stress test (Seahorse Biosciences) done using compounds which affect mitochondrial function, including oligomycin, FCCP, rotenone, and antimycin A.

### Electron-transport chain complex activities.

Electron-transport chain (ETC) complexes I, III, IV, and V (ATP synthase) activities were measured spectrophotometrically as previously described^29^. Briefly, brain and liver were resected from mice. Mitochondria were isolated from organs using BioVision Mammalian Mitochondria Isolation Kit for Tissue & Cultured Cells (Catalog #: K288), resuspended in 1 mL KME buffer each, and stored at −80 °C. Complex I activity was determined by measuring NADH oxidation at 340 nm. Complex III activity was assessed by measuring the reduction of cytochrome c at 550 nm in the presence of reduced decycloubiquinone (50 μM). Complex IV activity was evaluated by measuring the oxidation of cytochrome c at 550 nm. And ATP Synthase activity was measured using an assay coupled with pyruvate kinase, which converts ADP to ADP and produces pyruvate from phosphoenolpyruvate (PEP) measured at 340 nm. Each treatment group was comprised of 8 replicate wells in a 96-well plate. Bradford assay was used to measure protein concentration in isolated mitochondria from brain and liver that was used when performing calculations to obtain measurements expressed as nanomoles substrate converted/min/mg of protein. The mean measurement of each treatment group was compared with the mean of the control group (DMSO only) with a one-way ANOVA using GraphPad Prism 8 software program following the exclusion of outliers as determined by the ROUT (*Q* = 1%) outlier method.

### Dynamic light scattering.

Compounds were dissolved in DMSO to give a 10 mM stock concentration. This stock solution was diluted into PBS pH 7.4 to a final concentration of 20 μM, and the evaluated using a Malvern DLS Nano-ZS-90 system (Malvern Instruments).

### Iron–sulfur cluster release assay.

This method was adapted as previously described^9^. MitoNEET was prepared as described earlier and a stock of 4 mg/mL was used in the assay, in a 50 mM Tris buffer pH 8. In a clear UV transparent 96well plate, compounds were prepared as 10 mM stocks in DMSO, and added to the wells to give a final concentration of 20 μM. In each well 80 μL of the appropriate buffer, with either vehicle or compound, and then mitoNEET protein was added at 20 μL. The 96-well plate was analyzed in a BioTek Synergy 4 plate reader, reading at 458 nm for a period of time, at room temperature. For the pH 8, we used 50 mM Tris pH 8, with 100 mM NaCl, and for the pH 6.5, we used the 50 mM Bis–Tris pH 6.5 with 100 mM NaCl buffer solution.

### Capture assays.

A biotin–furosemide probe was prepared by first activating the carboxylic group of biotin with 1′-Carbonyldiimidazole (CDI) and conjugating it with furosemide. For the ELISA assay, streptavidin plates (Pierce) were incubated for 30 min with a dose-dilution of the probe, after which an equal amount of recombinant mitoNEET protein was placed into the wells. After 2 h incubation at 4 °C, the plate was washed with a wash buffer with PBS pH 7.4 containing 0.05% Tween-20. The amount of mitoNEET was determine using a mitoNEET primary antibody (ProteinTech) 1:1000, followed by secondary anti-rabbit horseradish peroxidase antibody 1:2000 for 1 h. Detection of the color was done at 450 nm after addition of 3,3′,5,5′-tetramethylbenzidine solution and stopping solutions (Pierce). For the capture assay, N2A cells were lysed and collected with radio-immunoprecipitation assay buffer. Recombinant mitoNEET was added to the 1.5 ml centrifuge tubes, and magnetic streptavidin Dynanbeads (Invitrogen) incubated with 100 μM furosemide probe was added to each tube. Furosemide, 0–100 μM was added to tubes and incubated for 30 min. At the end of the experiment, a magnetic tube rack was used to attract the magnetic beads. The solutions were washed with PBST (pH 7.4, 0.05% Tween-20), boiled for 5 min, and run on a BioRad 4–20% gel for Western Blot analysis (mitoNEET primary antibody 1:1000 and secondary HPR 1:2000).

### Synthesis of furosemide analogs 2.

A microwave vial containing 2,4-dichloro-5-sulfamoylbenzoic acid (**1**, 0.74 mmol), Et_3_N (2.9 mmol) and primary amine (1.85 mmol) in 2 mL of DME was heated for 5 h at 150 °C under microwave irradiation. The solution was cooled to room temperature, combined with EtOAc (30 mL) and washed twice with equal volumes of 5% citric acid and brine. The organic fraction was then dried over MgSO_4_, concentrated under reduced pressure, and loaded onto a silica gel column packed with 1:1 hexanes:EtOAc. Flash chromatography using 50–100% EtOAc in hexanes yielded pure furosemide analogs **2** as pale solids.

## Supplementary Material

SupplementaryMaterial_ChemComm2019

## Figures and Tables

**Table 1:** Data collection and refinement statistics

	mitoNEET-furosemide
*Data collection*	
Wavelength (Å)	1.74
Space group	*I* 4_1_ 2 2
Cell dimensions	
*a, b, c* (Å)	58.83, 58.83, 176.75
*α, β*, *γ* (°)	90, 90, 90
Resolution (Å)	30.30–1.95 (2.02–1.95)
*R*_sym_ or *R*_merge_	0.0353 (0.2533)
*I* /σ*I*	40.90 (2.24)
Completeness (%)	99.92 (100.00)
Redundancy	21.8 (21.1)
*Refinement*	
Resolution (Å)	30.30–1.95 (2.02–1.95)
No. of reflections	11791 (1146)
*R*_work_/*R*_free_	19.97/22.1
No. of atoms	689
Macromolecules	617
Ligand/ion	25
Solvent	47
*B-factors*	
Macromolecules	42.34
Ligand/ion	93.30
Solvent	41.38
*R.m.s. deviations*	
Bond lengths (Å)	0.010
Bond angles (°)	1.75

Values in parentheses are for highest-resolution shell.

**Table 2:** Displacement of [^3^H]rosiglitazone by sulfonamide compounds with recombinant human mitoNEET

Compounds	IC_50_ (μM) ±std error	clog*P*^a^
**Furosemide**	29.26 ±0.97	1.90
**1**	1021.00 ±0.96	1.11
**2a**	81.43 ±0.92	2.33
**2b**	42.56 ±1.03	2.86
**2c**	54.26 ±0.92	3.39
**2d**	9.12 ±1.04	3.92
**2e**	19.82 ±0.90	4.45
**2f**	6.24 ±0.88	4.98
**2k**	31.16 ± 1.02	3.31
**2l**	20.79 ± 1.01	2.72
**2n**	8.82 ± 1.00	2.87
**2p**	12.38 ± 1.23	2.37
**Arachidonic acid**	2.93 ± 1.06	6.3

a Calculate using Chemdraw 16.0.1.4
