# Supplementary material for: Crystal structure of the mitochondrial protein mitoNEET bound to a benze-sulfonide ligand
Source: Commun Chem. Author manuscript; Available in PMC 2020 May 7. (PMC7205193; doi:10.1038/s42004-019-0172-x)

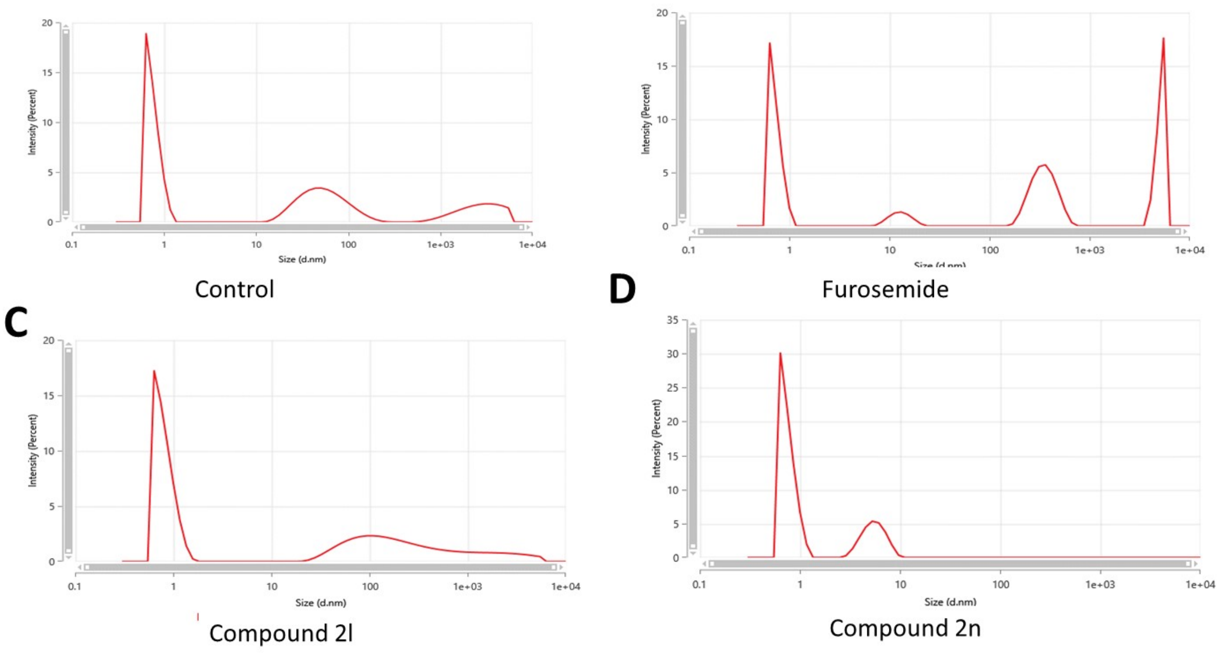

Supplementary Figure 1. Dynamic light scattering of the compounds tested. From the DLS reading, the compounds tested did not show any significant propensity to form aggregates.

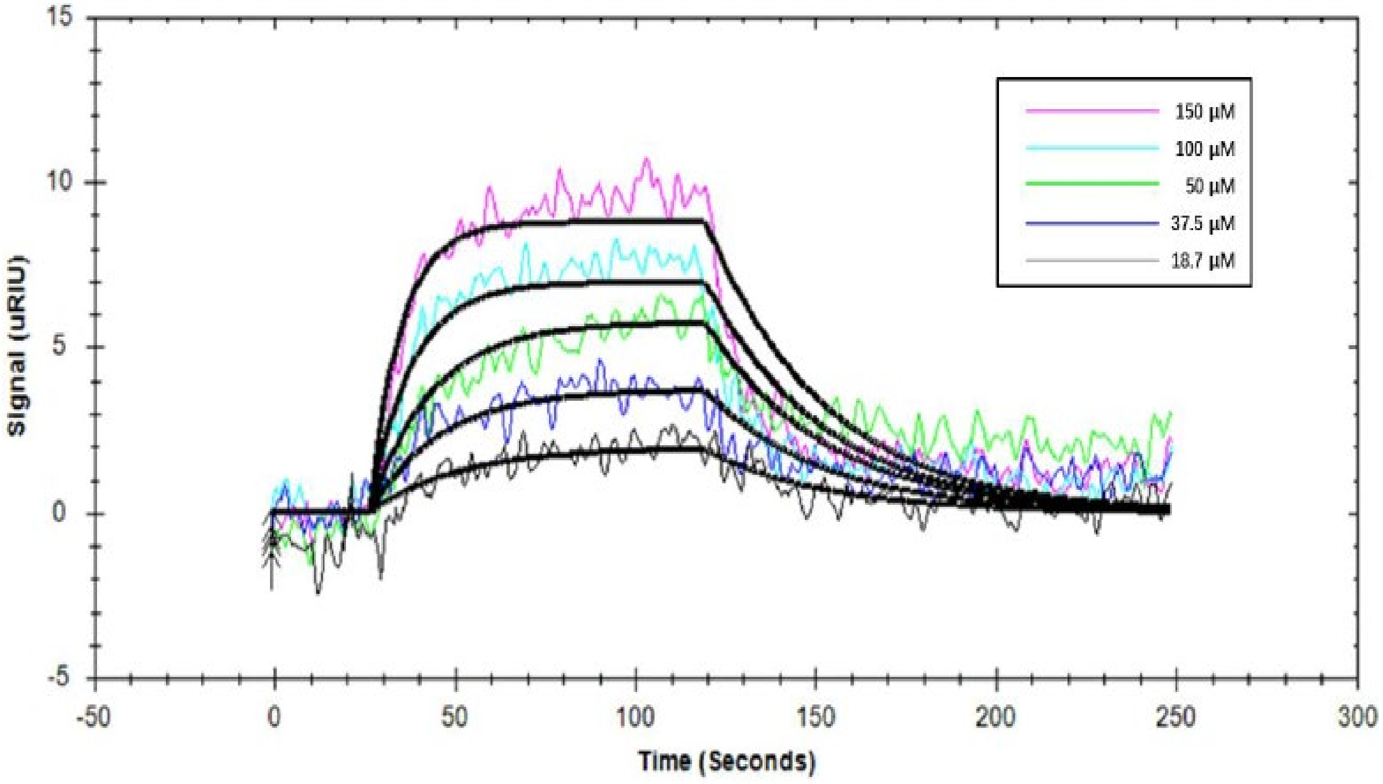

Supplementary Figure 2. Surface Plasmon Resonance of human recombinant mitoNEET interacting with furosemide. Recombinant human mitoNEET analyzed in the presence of several different concentrations of furosemide. Binding parameters were found to be an association rate constant *K*on of 5.61 x 10^2 M^{-1} s^{-1}, dissociation rate constant *K*off of 3.00 x 10^{-2} M^{-1} s^{-1} and the equilibrium dissociation constant *K*d is 53.5 µM.

Supplementary Methods

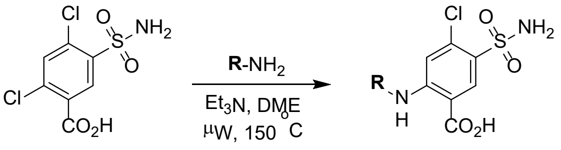

1 **2a-p**

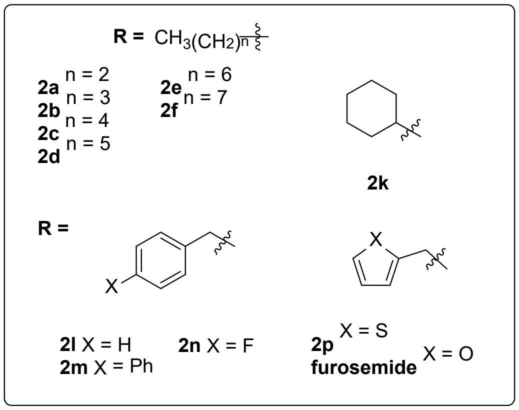

Synthesis of furosemide analogs 2. A microwave vial containing 2,4-dichloro-5-sulfamoylbenzoic acid (**1**, 0.74 mmol), Et_3N (2.9 mmol) and primary amine (1.85 mmol) in 2 mL of DME heated for 5 hours at 150 °C under microwave irradiation. The solution was cooled to room temperature, combined with EtOAc (30 mL) and washed twice with equal volumes of 5% citric acid and brine. The organic fraction was then dried over $MgSO_4$, concentrated under reduced pressure, and loaded onto a silica gel column packed with 1:1 hexanes:EtOAc. Flash chromatography using 50-100% EtOAc in hexanes yielded pure furosemide analogs **2** as pale solids.

4-chloro-2-(propylamino)-5-sulfamoylbenzoic acid (2a): mp 215-216 °C; TLC (SiO_2) R_f 0.21 (1:1 hexanes:EtOAc); ^{1}H NMR (400 MHz, Acetone-d_6) δ 11.23 (s, 1H), 8.59 (s, 1H), 8.40 (s, 1H), 6.93 (s, 1H), 6.52 – 6.46 (m, 1H), 3.30 (dtt, J = 8.4, 5.3, 3.1 Hz, 2H), 1.78 – 1.65 (m, 2H), 1.02 (td, J = 7.4, 1.4 Hz, 3H); ^{13}C NMR (101 MHz, Acetone-d_6) δ 11.8, 22.8, 45.2, 108.0, 114.0, 127.1, 135.1, 138.2, 154.5, 169.5; ESI-MS: m/z 291 $[M - H]^-$ (negative mode).

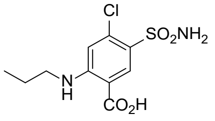

2a

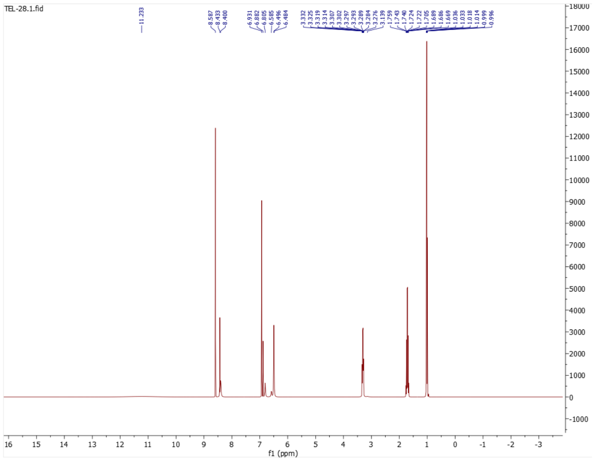

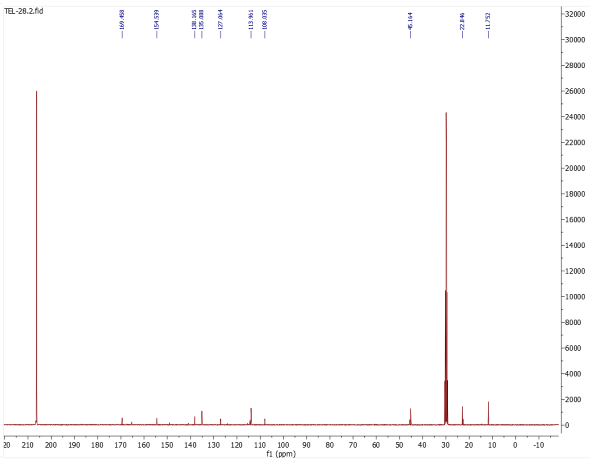

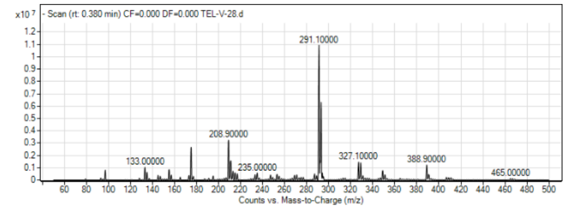

4-chloro-2-(butylamino)-5-sulfamoylbenzoic acid (2b): mp 208-210 °C; TLC (SiO_2) R_f 0.26 (1:1 hexanes:EtOAc); ^{1}H NMR (400 MHz, acetone-d_6) δ 11.45 (s, 1H), 8.59 (s, 1H), 6.94 (s, 1H), 6.49 (m, 2H), 3.38 – 3.29 (m, 2H), 1.74 – 1.63 (m, 2H), 1.54 – 1.40 (m, 2H), 0.97 (t, J = 7.3 Hz, 3H); ^{13}C NMR (101 MHz, acetone-d_6) δ 14.1, 20.9, 31.7, 43.2, 108.0, 114.0, 135.1, 138.2, 154.5, 169.4; ESI-MS: m/z 305 [M - H]- (negative mode).

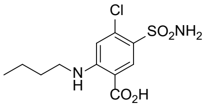

2b

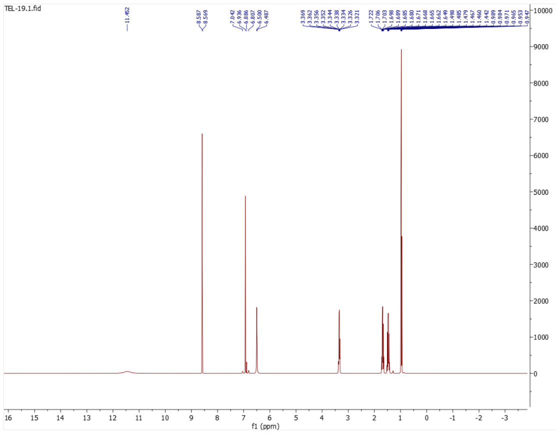

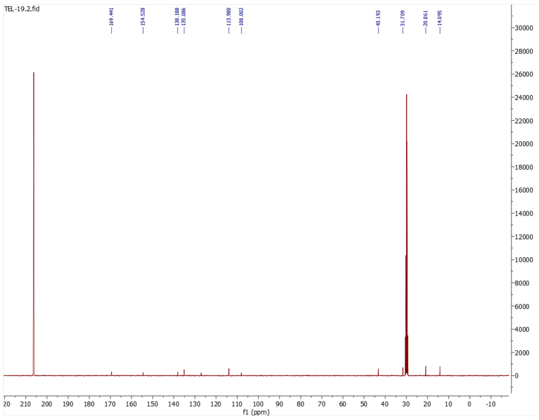

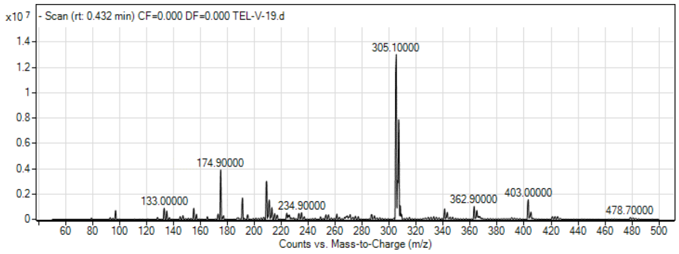

4-chloro-2-(pentylamino)-5-sulfamoylbenzoic acid (2c): mp 211-212 °C; TLC (SiO_2) R_f 0.29 (1:1 hexanes:EtOAc); ^{1}H NMR (400 MHz, Acetone-d_6) δ 8.59 (s, 1H), 6.93 (s, 1H), 6.62 – 6.31 (m, 1H), 3.33 (dtt, J = 9.2, 6.6, 3.8 Hz, 2H), 1.71 (h, J = 7.2, 6.8 Hz, 2H), 1.51 – 1.27 (m, 3H), 0.92 (t, J = 7.0 Hz, 3H); ^{13}C NMR (101 MHz, Acetone-d_6) δ 11.8, 22.8, 45.2, 108.0, 114.0, 127.1, 135.1, 138.2, 154.5, 169.5; ESI-MS: m/z 320 [M - H]$^-$ (negative mode).

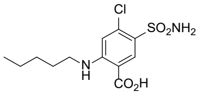

2c

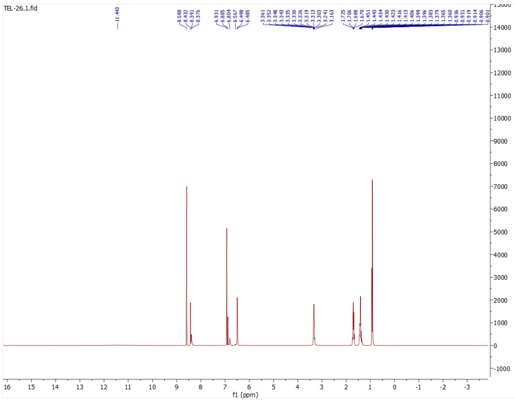

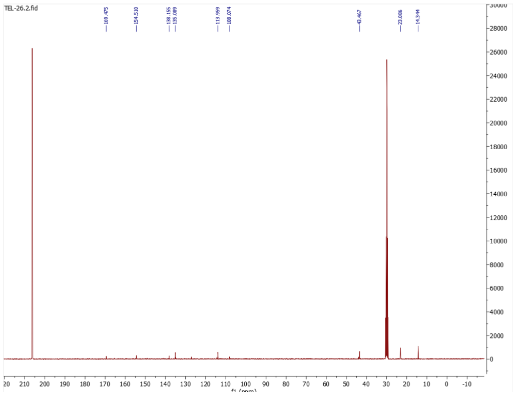

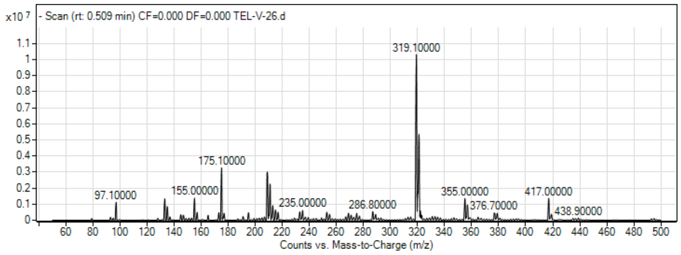

4-chloro-2-(hexylamino)-5-sulfamoylbenzoic acid (2d): mp 217-218 °C; TLC (SiO_2) R_f 0.29 (1:1 hexanes:EtOAc); ^{1}H NMR (400 MHz, Acetone-d_6) δ 8.59 (s, 1H), 8.45 – 8.17 (m, 1H), 6.93 (s, 1H), 6.57 – 6.43 (m, 1H), 3.34 (dtd, J = 7.0, 5.2, 2.6 Hz, 2H), 1.83 – 1.67 (m, 2H), 1.55 – 1.15 (m, 6H), 1.04 – 0.65 (m, 3H); ^{13}C NMR (101 MHz, Acetone-d_6) δ 14.3, 23.3, 27.4, 32.3, 43.5, 108.0, 114.0, 127.1, 135.1, 138.2, 154.5, 169.4; ESI-MS: m/z 333 [M - H]$^-$ (negative mode).

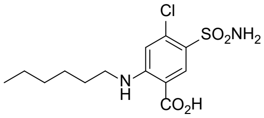

2d

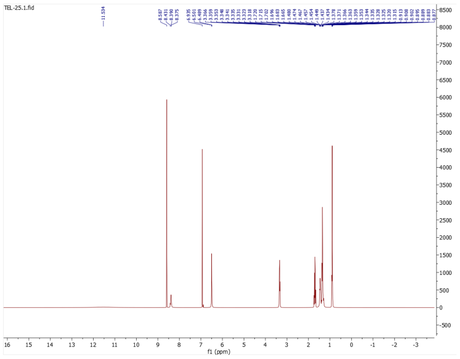

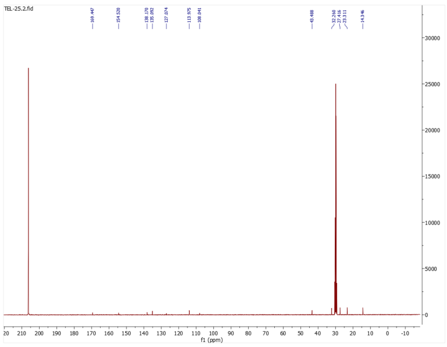

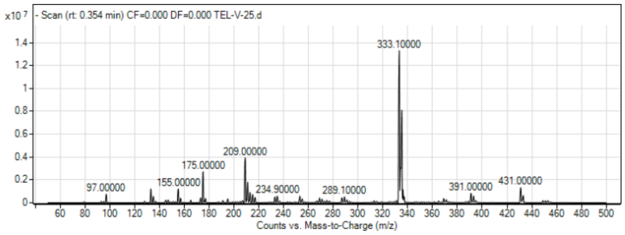

4-chloro-2-(heptylamino)-5-sulfamoylbenzoic acid (2e): mp 197-198 °C; TLC (SiO_2) R_f 0.35 (1:1 hexanes:EtOAc); ^{1}H NMR (400 MHz, DMSO-d_6) δ 13.23 (s, 1H), 8.38 (s, 1H), 8.28 (m, 1H), 7.31 (s, 2H), 6.90 (s, 1H), 3.25 (dt, J = 8.1, 3.6 Hz, 2H), 1.58 (h, J = 6.6 Hz, 2H), 1.39 – 1.22 (m, 9H); ^{13}C NMR (101 MHz, DMSO-d_6) δ 14.0, 22.0, 26.3, 28.2, 28.3, 31.2, 42.0, 107.4, 112.9, 126.0, 133.4, 136.4, 152.8, 168.8; ESI-MS: m/z 347 $[M - H]^-$ (negative mode).

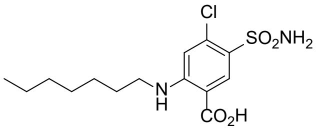

2e

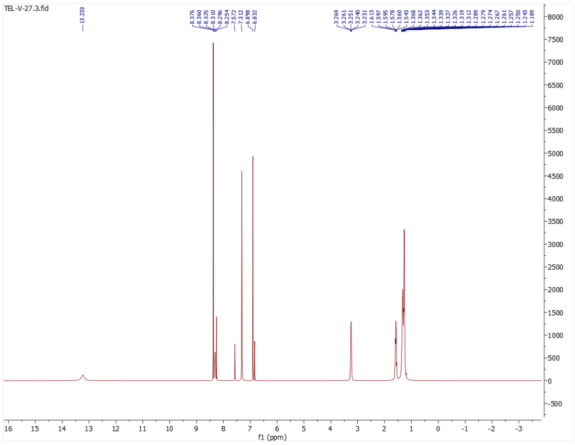

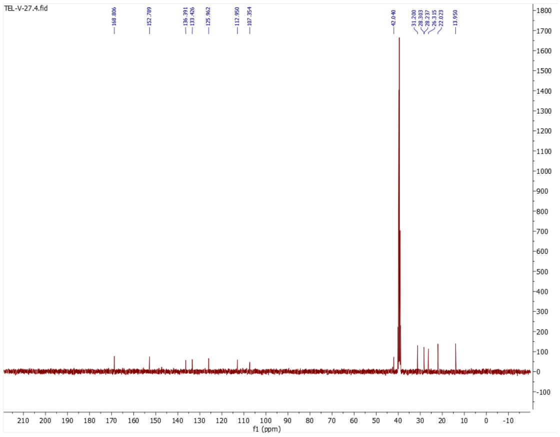

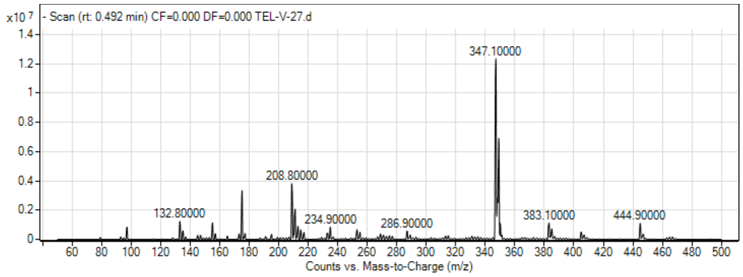

4-chloro-2-(octylamino)-5-sulfamoylbenzoic acid (2f): mp 184-185 °C; TLC (SiO_2) R_f 0.5 (1:1 hexanes:EtOAc); ^{1}H NMR (400 MHz, Acetone-d_6) δ 11.41 (s, 1H), 8.59 (s, 1H), 8.39 (s, 1H), 6.93 (s, 1H), 6.52 – 6.46 (m, 1H), 3.34 (dtd, J = 7.0, 5.2, 2.7 Hz, 2H), 1.71 (p, J = 7.2 Hz, 2H), 1.51 – 1.23 (m, 10H), 0.92 – 0.83 (m, 3H); ^{13}C NMR (101 MHz, Acetone-d_6) δ 14.4, 23.4, 27.7, 29.6, 30.0, 32.6, 43.5, 108.0, 114.0, 127.1, 135.1, 138.2, 154.5, 169.4; ESI-MS: m/z 361 [M - H]$^-$ (negative mode).

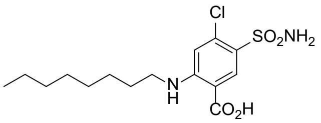

2f

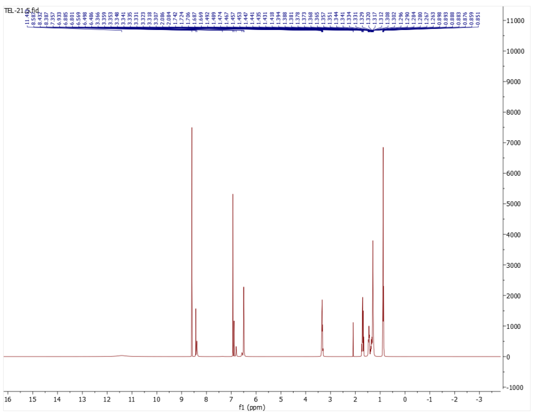

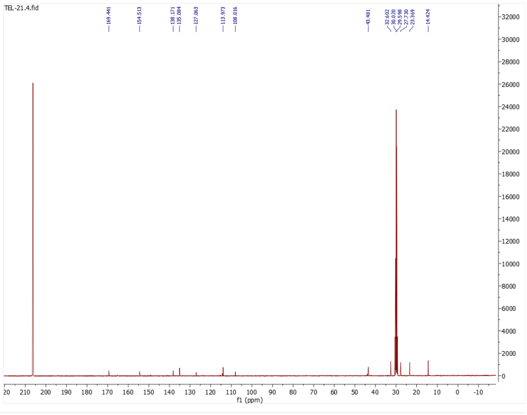

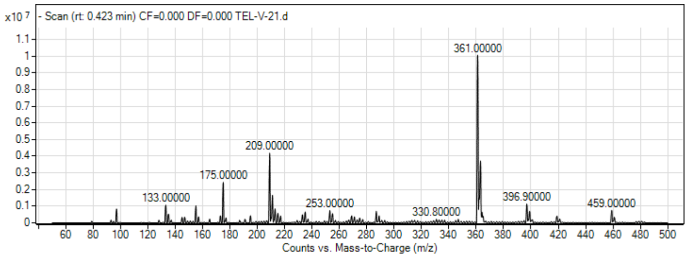

4-chloro-2-(cyclohexylamino)-5-sulfamoylbenzoic acid (2k): mp 105-108 °C; TLC (SiO_2) R_f 0.24 (1:1 hexanes:EtOAc); ^{1}H NMR (400 MHz, Acetone-d_6) δ 8.61 (s, 1H), 6.83 – 6.55 (m, 1H), 3.88 – 3.04 (m, 1H), 2.23 – 1.88 (m, 2H), 1.86 – 1.68 (m, 4H), 1.67 – 1.52 (m, 2H), 1.45 – 1.21 (m, 2H); ^{13}C NMR (101 MHz, Acetone-d_6) δ 25.3, 25.8, 26.6, 31.8, 32.7, 33.4, 50.9, 58.9, 112.8, 125.5, 135.4, 153.2, 172.3; ESI-MS: m/z 331 [M - H]$^-$ (negative mode).

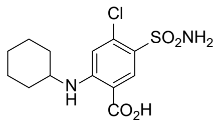

2k

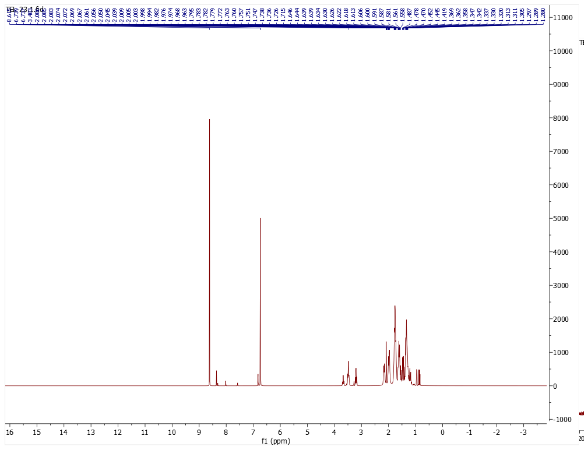

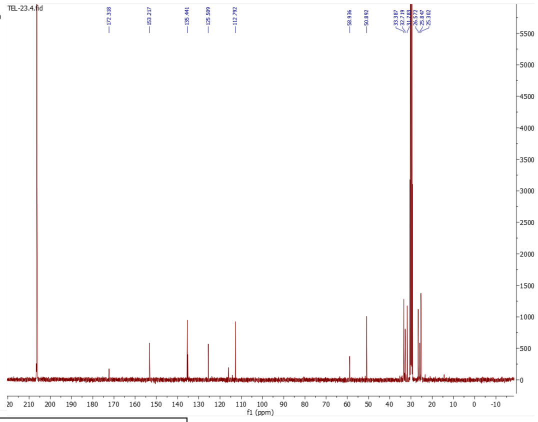

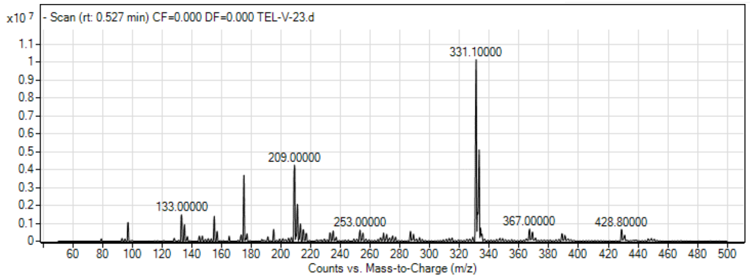

2-(benzylamino)-4-chloro-5-sulfamoylbenzoic acid (2l): mp 229-230 °C; TLC (SiO_2) R_f 0.31 (1:1 hexanes:EtOAc); ^{1}H NMR (400 MHz, Acetone-d_6) δ 11.52 (bs, 1H), 8.83 (s, 1H), 8.62 (s, 1H), 7.48 – 7.41 (m, 1H), 7.45 – 7.35 (m, 3H), 7.39 – 7.29 (m, 1H), 7.33 – 7.25 (m, 1H), 6.91 (s, 1H), 6.54 – 6.48 (m, 1H), 4.67 – 4.61 (m, 2H); ^{13}C NMR (101 MHz, Acetone-d_6) δ 47.3, 108.7, 114.6, 128.2, 128.4, 129.7, 135.0, 138.0, 139.1, 154.3, 169.3; ESI-MS: *m*/*z* 339 [M - H]$^-$ (negative mode).

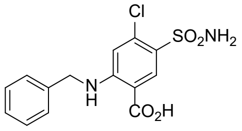

2l

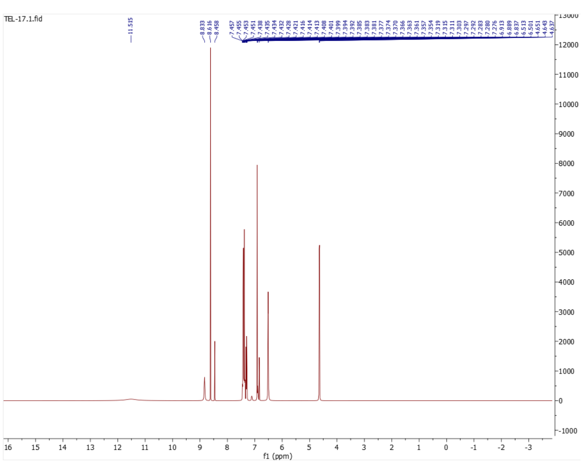

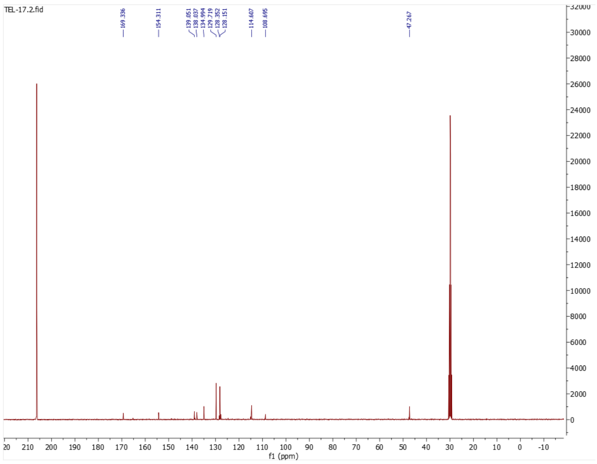

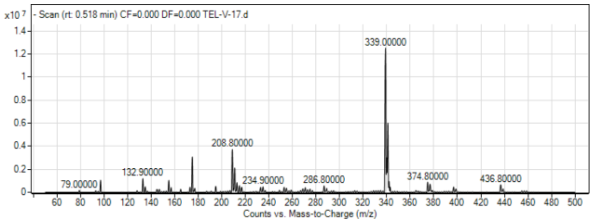

4-chloro-2-((4-fluorobenzyl)amino)-5-sulfamoylbenzoic acid (2n): mp 215-216 °C; TLC (SiO_2) R_f 0.32 (1:1 hexanes:EtOAc); ^{1}H NMR (400 MHz, Acetone-d_6) δ 11.52 (s, 1H), 8.83 (s, 1H), 8.61 (s, 1H), 7.54 – 7.42 (m, 2H), 7.14 (td, J = 8.9, 2.2 Hz, 2H), 6.89 (d, J = 3.8 Hz, 1H), 6.53 (s, 1H), 4.68 – 4.61 (m, 2H); ^{13}C NMR (101 MHz, Acetone-d_6) δ 46.5, 108.8, 114.6, 115.0, 116.5, 127.8, 130.1, 135.0, 138.0, 154.2, 161.8, 164.3, 169.4; ESI-MS: m/z 357 [M - H]$^-$ (negative mode).

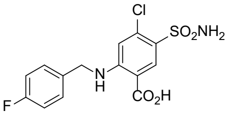

2n

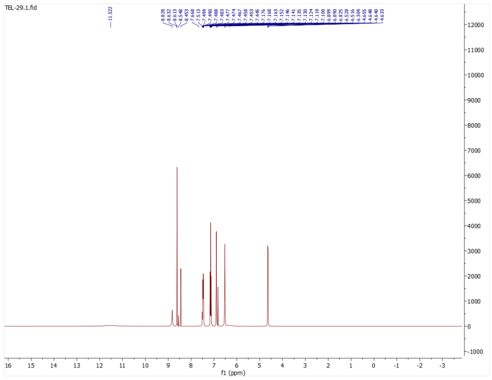

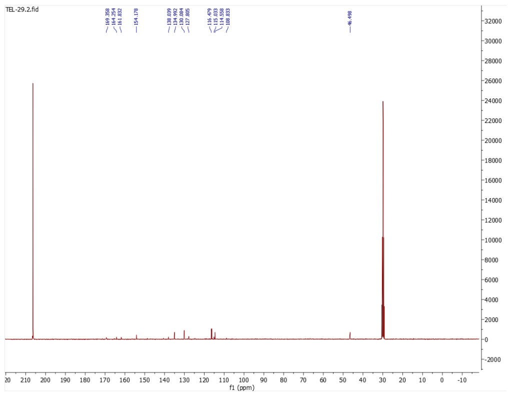

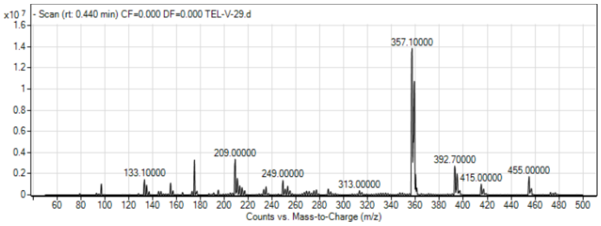

4-chloro-5-sulfamoyl-2-((thiophen-2-ylmethyl)amino)benzoic acid (2p): mp 271-273 °C (decomp.); TLC (SiO_2) R_f 0.24 (1:1 hexanes:EtOAc); ^{1}H NMR (400 MHz, Acetone-d_6) δ 11.32 (bs, 1H), 8.83 (s, 1H), 8.62 (d, J = 3.3 Hz, 1H), 7.38 (d, J = 4.5 Hz, 1H), 7.16 (d, J = 4.2 Hz, 1H), 7.08 – 6.97 (m, 2H), 6.52 (s, 1H), 4.85 (t, J = 4.6 Hz, 2H); ^{13}C NMR (101 MHz, Acetone-d_6) δ 42.5, 108.9, 114.7, 126.1, 126.7, 128.0, 135.0, 138.1, 142.3, 153.9, 169.2; ESI-MS: m/z 345 [M - H]$^-$ (negative mode).

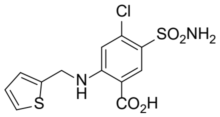

2p

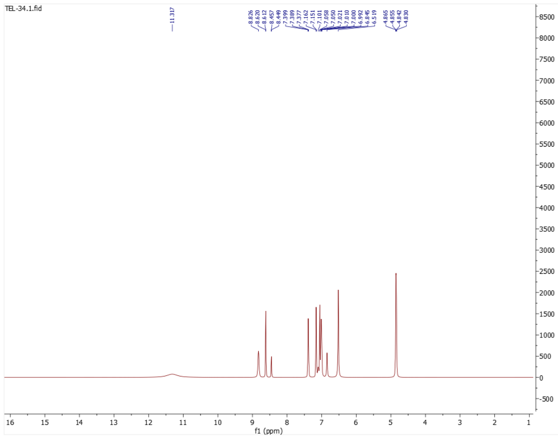

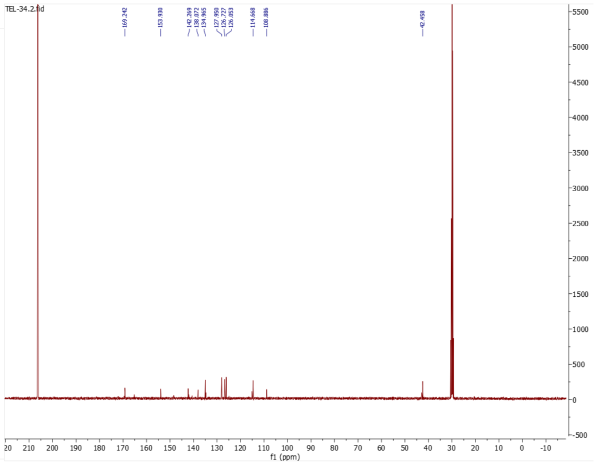

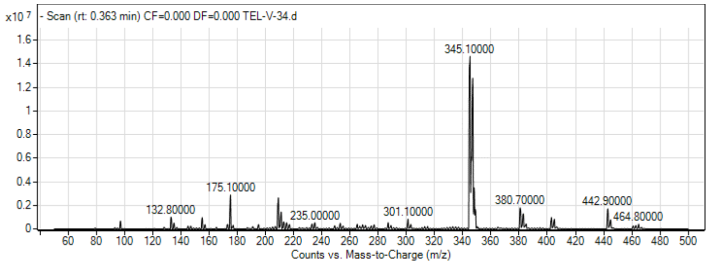

Supplement: SupplementaryMaterial_ChemComm2019 [file NIHMS1572889-supplement-SupplementaryMaterial_ChemComm2019.pdf]
